# Prevalence and Associated Factors of Polypharmacy Among Emirati Community-Dwelling Older Adults: A Cross-Sectional Survey

**DOI:** 10.3390/healthcare14050648

**Published:** 2026-03-04

**Authors:** Fatma M. Ibrahim, Tarun Wadhwa, Mona Gamal Mohamed, Eman Abdelaziz Rashad Dabou, Amal Mohamed, Khaled Elbarbary

**Affiliations:** 1 Faculty of Nursing, Mansoura University, Mansoura 35516, Egypt; 2 RAK College of Nursing, RAK Medical and Health Sciences University, Ras Al Khaimah 11172, United Arab Emirates; 3 RAK College of Pharmacy, RAK Medical and Health Sciences University, Ras Al Khaimah 11172, United Arab Emirates; 4 Faculty of Nursing, Alexandria University, Alexandria 21527, Egypt; 5 Faculty of Medicine, Mansoura University, Mansoura 35516, Egypt

**Keywords:** polypharmacy, hyper-polypharmacy, older adults, activities of daily living, United Arab Emirates

## Abstract

**Highlights:**

**What are the main findings?**

- Polypharmacy (5–9 medications) and hyper-polypharmacy (≥10 medications) were highly prevalent among Emirati community-dwelling older adults attending primary care in Ras Al Khaimah.
- Hyper-polypharmacy was associated with poorer functional status (lower Katz Index of Independence in Activities of Daily Living and Lawton Instrumental Activities of Daily Living scores).

**What are the implications of the main findings?**

- Routine medication review and deprescribing should be integrated into primary care for older adults, prioritizing individuals with functional limitations.
- Use of non-prescribed products (OTC medicines, supplements, and herbal/traditional remedies) was common and should be explicitly documented and addressed during medication review.

**Abstract:**

**Background/Objectives:** Older adults are vulnerable to inappropriate prescribing and polypharmacy, yet data from the United Arab Emirates are scarce. We estimated the prevalence and correlates of polypharmacy and hyper-polypharmacy among Emirati community-dwelling older adults. **Methods**: A cross-sectional study of 200 Emiratis aged ≥60 years registered at primary health centers in Ras Al Khaimah used convenience sampling. Data on chronic conditions, medications, and function (Katz Index of Independence in Activities of Daily Living [Katz ADL]; Lawton Instrumental Activities of Daily Living scale [Lawton IADL]) were collected using a structured Arabic questionnaire. Polypharmacy was defined as 5–9 and hyper-polypharmacy as ≥10 medications. **Results:** Overall, 60% used 5–9 and 40% ≥10 medications; 90% had ≥1 chronic disease and 83% used non-prescribed drugs, commonly analgesics, vitamins, and laxatives. Higher medication burden was associated with poorer ADL and IADL. Younger age and lower Katz ADL scores predicted hyper-polypharmacy. **Conclusions:** Polypharmacy is highly prevalent and linked to functional limitations, supporting routine medication review, deprescribing, and monitoring of non-prescribed use.

## 1. Introduction

As the global population ages, the number of people aged 60 years and older is projected to almost double between 2015 and 2050, rising from 12% to 22% of the world’s population [1]. With increasing age, the prevalence of chronic conditions grows, and many older adults are treated with multiple medications simultaneously, a practice commonly referred to as polypharmacy [2]. Although pharmacotherapy is fundamental to the management of chronic diseases, taking many medications at once can lead to substantial harm [3].

Polypharmacy does not have a single universally accepted definition, but it is frequently described as the daily use of five or more medications, while the term hyper-polypharmacy is often reserved for the use of ten or more medications [4]. Older adults are particularly susceptible to adverse effects from multiple medications due to age-related changes in cognition, pharmacokinetics, and pharmacodynamics, including altered drug metabolism and reduced renal clearance [5,6]. As the number of prescribed medications increases, so does the risk of drug–drug interactions, adverse drug reactions (ADRs), poor adherence, and even mortality [7].

The burden of polypharmacy is increasingly recognized worldwide. Studies have reported polypharmacy prevalence estimates of approximately 65.1% in the United States [7], 50.1% in China [8], and between 26.3% and 39.9% in various European countries [9]. A meta-analysis of 24 studies involving nearly 3 million participants showed that taking ≥5 medications was associated with a 1.28-fold increase in mortality risk, while taking ≥10 medications increased mortality risk 1.44-fold [7]. ADR risk rises steeply with the number of medications, with estimates reaching 58% when five drugs are used and 82% when more than seven are used [10]. Commonly implicated drug classes in preventable drug-related hospital admissions include antiplatelets, diuretics, nonsteroidal anti-inflammatory drugs (NSAIDs), and anticoagulants [11].

The clinical manifestations of polypharmacy are often misattributed to normal aging, including fatigue, somnolence, decreased alertness, gastrointestinal disturbances, falls, confusion, mood changes, tremors, weakness, hallucinations, and dizziness [12]. Polypharmacy has been identified as an independent risk factor for hip fractures in older adults [13] and is consistently associated with falls, frailty, cognitive impairment, and functional decline [9,14,15,16,17,18].

Evaluating polypharmacy in older adults is therefore critical to prevent potential harm. Interdisciplinary medication review, deprescribing of unnecessary or inappropriate drugs (e.g., guided by the Beers Criteria), and regular assessment of treatment goals can reduce risks and improve patient safety [9,14,19]. Collaborative care involving physicians, pharmacists, and nurses, supported by electronic health records, can enhance medication monitoring and deprescribing efforts [14,19].

In the United Arab Emirates (UAE), the aging population is projected to increase substantially, contributing to a growing burden of chronic disease and polypharmacy. Yet there is a notable lack of localized evidence on the prevalence and determinants of polypharmacy among Emirati community-dwelling older adults. Existing studies from the Gulf region suggest that polypharmacy is common, but most have focused on mixed or non-national populations and hospital-based samples [20,21,22,23].

This study addresses this gap by examining polypharmacy exclusively among older Emirati nationals living in the community and registered with primary health centers. Specifically, we aimed to (1) estimate the prevalence of polypharmacy and hyper-polypharmacy within this cohort and (2) identify sociodemographic, clinical, and functional factors associated with higher medication burden. The findings are intended to inform national strategies for medication review, deprescribing, and safer pharmacotherapy in older adults in the UAE.

## 2. Materials and Methods

### 2.1. Study Design and Setting

We conducted a descriptive cross-sectional study from March 2025 to May 2025 in Ras Al Khaimah (RAK), one of the seven emirates of the United Arab Emirates. RAK was selected using simple random sampling. Participants were recruited from two primary healthcare centers providing home-care services to older adults: Julphar and Ras Al Khaimah Healthcare Centers.

Data collection was coordinated with the home-care teams at the participating primary healthcare centers. Potentially eligible older adults were identified from the centers’ home-care service lists and/or scheduled visits during the study period, and trained members of the research team (external to routine clinical care) approached them to explain the study in Arabic and obtain written informed consent.

Data were collected through face-to-face, structured interviews conducted in Arabic in a private setting at the primary health center or during home-care visits. Because medication verification was a core component of the protocol and many participants had limited digital access, an online survey option was not used. When appropriate, family members/caregivers could assist with practical aspects of the visit (e.g., retrieving medication containers) while responses were obtained from participants.

### 2.2. Participants and Eligibility Criteria

The study population included community-dwelling Emirati adults aged 60 years and older who were:

- Registered at one of the participating primary health centers.
- Residents of the United Arab Emirates.
- Able to communicate and participate in an interview.
- Willing to provide written informed consent.

Exclusion criteria were:

- A medical diagnosis of dementia, Alzheimer’s disease, or moderate-to-severe cognitive impairment.
- Severe physical impairment causing complete dependence in basic self-care and instrumental activities.

Cognitive status was screened using clinical records and interviewer judgment. Individuals who could not participate in two-way verbal exchanges or comprehend and execute simple instructions were excluded. Those with moderate-to-severe dementia (e.g., Mini-Mental State Examination ≤ 10) or with behavioral features that jeopardized informed consent or data validity were not enrolled. Severe physical impairment was characterized by marked dependence in basic self-care and instrumental tasks, as indicated by low Katz ADL and Lawton IADL scores. These exclusions were necessary to ensure valid informed consent and reliable participation in the interview and medication verification process; however, they may have under-represented the most cognitively/physically impaired older adults who could be at higher risk of polypharmacy and hyper-polypharmacy. This potential selection bias is considered in the limitations.

### 2.3. Sampling Strategy and Sample Size

Because no complete registry of Emirati older adults is available and logistic constraints precluded probability sampling, we used convenience non-probability sampling. Older adults meeting the inclusion criteria and attending or registered with the two centers’ home-care services during the study period were invited to participate. This approach is consistent with STROBE recommendations to transparently report analytical methods and potential biases related to non-probability sampling.

A priori sample size calculations were conducted using Epi Info 7 (Centers for Disease Control and Prevention, Atlanta, GA, USA), assuming α = 0.05 and 80% power. A minimum of 175 participants was required to detect an odds ratio (OR) ≥ 2.0 for the main comparison. To account for potential non-response and incomplete data (up to 12%) and to increase the precision of our estimates, we increased the target sample size to 200 participants. A post hoc sensitivity analysis indicated that with N = 200, the study retained > 80% power to detect ORs > 1.9 at α = 0.05.

### 2.4. Questionnaire Development and Cross-Cultural Adaptation

The questionnaire underwent rigorous cross-cultural adaptation following established international guidelines. Because Arabic is the primary language of the Emirati population, we used a systematic forward-backward translation process conducted by bilingual experts fluent in both English and Arabic, in line with ISPOR recommendations for linguistic and cultural adaptation of instruments [24].

To ensure contextual relevance, culturally sensitive modifications were made to reflect local health beliefs and practices. For example, items were added on the use of traditional remedies commonly used in the UAE, and wording was adapted to match local colloquial expressions and healthcare-seeking patterns among older Emiratis.

Face validity was evaluated by an expert panel comprising three geriatricians and two pharmacists familiar with geriatric care in the UAE. Content validity was assessed using their structured feedback on item relevance and clarity. Cognitive interviews were then conducted with 10 older adults to assess comprehension, cultural appropriateness, and acceptability. These pilot participants were not included in the final analysis. An English-language version of the final questionnaire is available as Supplementary File S1.

### 2.5. Measures

#### 2.5.1. Tool I: Sociodemographic and Clinical Characteristics

This section captured data on gender, age and age category, marital status, educational level, current employment, monthly income, living arrangement, smoking status, life satisfaction, perceived health status, presence and type of chronic diseases, and history of falls in the last 12 months.

#### 2.5.2. Tool II: Medication Use and Polypharmacy

Medication use was assessed by asking participants about all regularly used medications, including:

- Prescribed drugs;
- Over-the-counter (OTC) medications;
- Herbal remedies, and;
- Dietary supplements.

We included non-prescribed products (e.g., vitamins/supplements and herbal/traditional remedies) in the medication count because they contribute to overall regimen complexity and may still cause toxicity or clinically relevant interactions with prescription medicines. To aid interpretation, we also reported non-prescribed product use separately by type.

To reduce recall bias, a structured “brown-bag” medication review was performed at each interview. Participants were asked in advance to bring all medications they were currently using, including prescription medicines as well as non-prescribed products (OTC medicines, vitamins/supplements, and herbal/traditional remedies), preferably in their original containers (or as written lists when containers were unavailable). Research staff reviewed each item with the participant and recorded the medicine name (brand/generic), dose/strength, dosage form, and frequency; prescription status (prescribed vs. self-initiated) was also noted when possible using participant report and available primary-care/pharmacy records. Each distinct medicinal product used regularly at the time of the interview (including fixed-dose combination products) was counted as one medication for the purpose of the medication-count definition.

Based on the total number of medications used regularly, participants were categorized into:

- Polypharmacy: 5–9 medications.
- Hyper-polypharmacy: ≥10 medications.

These cut-offs are consistent with widely used thresholds in geriatric research [4,7]. In addition, we documented the use and types of non-prescribed medications (e.g., analgesics, vitamins, laxatives). Multi-item scales pertaining to perceived health status and life satisfaction demonstrated acceptable internal consistency (Cronbach’s alpha > 0.70).

#### 2.5.3. Tool III: Katz Index of Independence in Activities of Daily Living (Katz ADL)

Functional dependence in basic activities of daily living was evaluated using the Katz Index [25,26]. The scale includes six ADL domains:

- Bathing.
- Dressing.
- Toileting.
- Transferring.
- Continence.
- Feeding.

Each item was scored 1 = independent or 0 = dependent, and scores were summed to yield a total score from 0 to 6. Higher scores indicate greater independence. For descriptive purposes, we classified participants as:

- Independent: total score = 6.
- Partially dependent: total score 3–5.
- Totally dependent: total score 0–2.

In our sample, the Katz Index showed excellent internal consistency (Cronbach’s alpha = 0.966).

#### 2.5.4. Tool IV: Lawton Instrumental Activities of Daily Living (Lawton IADL) Scale

Instrumental activities of daily living were assessed using the Lawton IADL Scale [20], which encompasses eight domains:

- Using the telephone.
- Shopping.
- Food preparation.
- Housekeeping.
- Laundry.
- Transportation.
- Responsibility for medications.
- Ability to handle finances.

Each item was scored 1 = independent or 0 = dependent, yielding a total score ranging from 0 to 8. To classify overall IADL status, total scores were converted into percentages and categorized as:

- Dependent: 0–<25%.
- Partially dependent: 25–<75%.
- Independent: ≥75%.

The scale demonstrated good reliability in this study (Cronbach’s alpha = 0.85).

### 2.6. Ethical Considerations

The study followed the ethical principles of the Declaration of Helsinki. Ethical approval was obtained from the Ministry of Health and Prevention Research Ethics Committee, United Arab Emirates (MOHAP/REC/2024/69-2024-F-N; approval date: 4 March 2025). Written informed consent was obtained from all participants before data collection. Participants’ privacy and confidentiality were assured, and data were anonymized for analysis.

### 2.7. Analytical Strategy

Data were coded, entered, and analyzed using IBM SPSS Statistics, version 22 (SPSS Inc., Chicago, IL, USA). Categorical variables were summarized as frequencies and percentages. Continuous variables were described using means and standard deviations where approximately normally distributed, or medians and interquartile ranges where appropriate.

Group differences in sociodemographic, clinical, and functional characteristics by medication-burden category (polypharmacy vs. hyper-polypharmacy) were examined using:

- Chi-square (χ^2^) tests for categorical variables, and
- Independent-samples *t* tests or non-parametric equivalents (e.g., Mann–Whitney U) for continuous variables, as appropriate.

To identify independent predictors of higher medication burden, we fitted Firth’s penalized likelihood logistic regression model, with hyper-polypharmacy (≥10 medications) vs. polypharmacy (5–9 medications) as the dependent variable. Firth’s method was chosen a priori to reduce small-sample bias and to obtain finite estimates in the presence of (quasi-)separation. Alternative penalized approaches (e.g., ridge/L2 logistic regression) can also stabilize estimates but require selection of a tuning parameter; therefore, Firth’s approach was preferred for interpretability in this applied setting. Because the outcome was not rare in our sample, modifications of Firth regression that primarily target probability calibration in rare-event settings (e.g., FLIC/FLAC) were not applied. Candidate predictors were selected a priori based on clinical relevance and prior literature and included:

- Age category (60–74, 75–84, ≥85 years; entered as an ordinal variable);
- Gender;
- Marital status;
- Educational level;
- History of falls;
- Smoking status;
- Presence of chronic diseases and selected disease categories;
- Total Katz ADL score;
- Use of non-prescribed medications and specific medication types.

Multicollinearity was assessed using variance inflation factors (VIFs); all included variables had VIF < 5, indicating no serious multicollinearity. Missing data were minimal (<5% per variable), and complete-case analysis (listwise deletion) was used in the regression models. Sensitivity checks indicated that excluding cases with missing data did not materially alter the direction or magnitude of the main associations.

Results from the logistic regression are presented as odds ratios (ORs) with 95% confidence intervals (CIs). A two-sided *p*-value < 0.05 was considered statistically significant.

## 3. Results

### 3.1. Sociodemographic Characteristics and Perceived Health

A total of 200 Emirati older adults participated in the study. The majority were female (58.0%). Most participants were married (73.0%), currently working (71.0%), and living with their families (79.0%) (Table 1).

In terms of age distribution, 44.0% were aged 60–74 years, 50.0% were 75–84 years, and 6.0% were aged ≥85 years (Table 1).

### 3.2. Chronic Conditions and History of Falls

Most participants (90.0%) reported at least one chronic disease (Table 2). Diabetes mellitus (85.0%), orthopedic diseases (83.0%), kidney diseases (77.0%), and cardiovascular diseases (69.0%) were the most common conditions.

**Figure 1:**
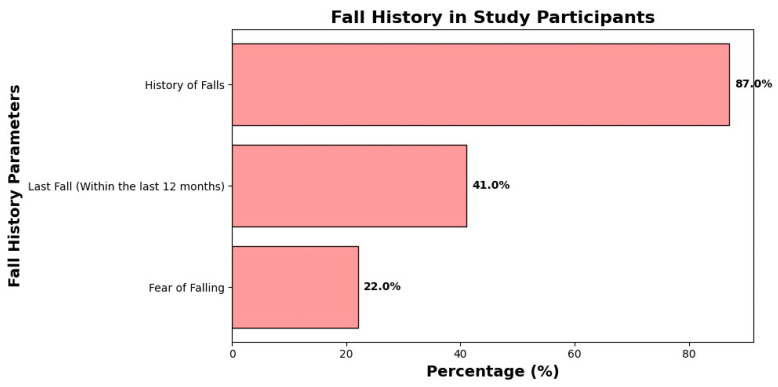
History of falls distribution in study participants.

Falls were frequent: 87.0% of participants reported a history of falls, and 41.0% had experienced a fall within the previous 12 months. Fear of falling was reported by 22.0% of participants (Figure 1).

### 3.3. Medication and Polypharmacy Patterns

All participants were taking at least five medications at the time of the study. Of them, 60.0% (n = 120) had polypharmacy (5–9 medications) and 40.0% (n = 80) had hyper-polypharmacy (≥10 medications) (Table 3).

Use of non-prescribed medications was also common: 83.0% reported using at least one non-prescribed medication. Analgesics were used by 83.0% of participants, vitamins by 77.0%, laxatives by 71.0%, and other non-prescribed agents by 59.0% (Table 3).

### 3.4. Functional Status: ADL (Katz Index)

Table 4 shows the association between medication burden and Katz ADL scores. Participants in the hyper-polypharmacy group had markedly lower mean scores across all six ADL items (bathing, dressing, toileting, transferring, continence, and feeding) than those in the polypharmacy group (all *p* < 0.001).

The mean total Katz score was 5.92 ± 0.28 in the polypharmacy group and 1.35 ± 1.60 in the hyper-polypharmacy group (*p* < 0.001). Almost all participants in the polypharmacy group were classified as independent in ADLs (91.7%), whereas 60.0% of those in the hyper-polypharmacy group were totally dependent and 40.0% partially dependent (Table 4).

### 3.5. Functional Status: IADL (Lawton Scale)

Similarly, significant differences were observed in IADL performance (Table 5). Participants with hyper-polypharmacy had substantially lower mean scores in all eight Lawton IADL domains and lower total IADL scores than those with polypharmacy (all *p* < 0.001).

The mean total Lawton score was 7.78 ± 0.45 in the polypharmacy group and 1.77 ± 2.40 in the hyper-polypharmacy group. All participants in the polypharmacy group were classified as IADL-independent, whereas 65.0% of those in the hyper-polypharmacy group were totally dependent and 17.5% partially dependent (Table 5).

### 3.6. Predictors of Higher Medication Burden (Firth Logistic Regression)

Firth’s penalized logistic regression was used to identify predictors of hyper-polypharmacy vs. polypharmacy (Table 6). After adjustment for potential confounders, two variables remained statistically significant:

- Age category (per category increase): OR 0.34 (95% CI 0.15–0.78; *p* = 0.011).
- Total Katz ADL score: OR 0.07 (95% CI 0.01–0.43; *p* = 0.004).

These results indicate that younger age and lower ADL independence were associated with higher odds of being in the hyper-polypharmacy group. In contrast, gender, marital status, educational level, history of falls, smoking status, chronic disease burden, and use of non-prescribed medications were not statistically significant independent predictors in the adjusted model.

Regression results are summarized in Table 6.

## 4. Discussion

This study provides new evidence on polypharmacy and hyper-polypharmacy among community-dwelling Emirati older adults registered in primary care in Ras Al Khaimah. We found that all participants were taking at least five medications, with 60% classified as having polypharmacy (5–9 medications) and 40% as having hyper-polypharmacy (≥10 medications). Most participants had multiple chronic conditions, and use of non-prescribed medications, particularly analgesics, vitamins, and laxatives, was highly prevalent. Importantly, higher medication burden was strongly associated with greater functional dependence in both basic and instrumental activities of daily living.

### 4.1. Polypharmacy Prevalence in Regional and International Context

Our observed distribution of polypharmacy and hyper-polypharmacy suggests a very high medication burden in this cohort of older Emiratis. Studies from the Gulf Cooperation Council (GCC) region have reported varied polypharmacy prevalences, reflecting different settings and definitions. For example, a Saudi study among adult medical outpatients reported that 13.1% had polypharmacy defined as ≥4 medications [22], while a community-based study in Kuwait found a prevalence of 58.4% for polypharmacy (5–8 medications) and 10.2% for excessive polypharmacy (>8 medications) among older adults [23]. An Egyptian study among elderly individuals reported that 85.3% used more than five medications [27].

Compared with European and North American data [7,8,9,16,27,28,29,30], the medication burden in our sample appears high. This may reflect a combination of factors, including high multimorbidity, frequent specialist consultations, fragmented care, cultural preferences for pharmacological treatment, and relatively easy access to OTC and non-prescribed medications. Our study adds to this literature by focusing exclusively on Emirati nationals and linking medication burden to functional status [31]

### 4.2. Multimorbidity, Self-Medication, and Medication Burden

The vast majority of participants (90%) reported at least one chronic condition, and many had multiple conditions such as diabetes mellitus, cardiovascular disease, kidney disease, and orthopedic problems. This pattern is consistent with previous work showing a strong association between multimorbidity and polypharmacy [15,18,32]. As the number of chronic conditions increases, the likelihood of being prescribed multiple drugs, and thus the risk of ADRs and drug–drug interactions-rises substantially [7,9,15,16].

Self-medication was very common in our cohort, with 83% of participants using non-prescribed medications. Analgesics, vitamins, and laxatives were the most frequently reported, similar to patterns seen in other countries [16]. While some OTC use can be appropriate, unsupervised self-medication can exacerbate polypharmacy, raise the risk of ADRs, and complicate deprescribing efforts. This highlights the need for stronger public education and better integration of OTC and traditional remedies into medication review processes.

### 4.3. Functional Status and Medication Burden

A key finding of this study is the strong association between higher medication burden and functional dependence. Older adults in the hyper-polypharmacy group had markedly lower Katz ADL and Lawton IADL scores than those in the polypharmacy group, with large differences across all individual ADL and IADL domains. This is consistent with previous evidence that polypharmacy is linked to functional impairment, frailty, and loss of independence [9,15,16,17,18].

In the Firth logistic regression, lower ADL independence (lower Katz scores) was independently associated with higher odds of hyper-polypharmacy. This suggests a potentially bidirectional relationship: on the one hand, functionally dependent individuals may require more medications for symptom control and comorbidity management; on the other hand, inappropriate or excessive medication use may in turn worsen function by causing side effects such as dizziness, sedation, and orthostatic hypotension [9,14,17]. Although our cross-sectional design cannot establish causality, the strong association underscores the importance of integrating medication review into comprehensive geriatric assessment.

### 4.4. Age and Medication Burden

Interestingly, younger age within this older cohort was associated with higher odds of hyper-polypharmacy, while older age appeared protective in the adjusted model. This is reflected in the OR of 0.34 for age, indicating that each increment in age (as coded) was associated with lower odds of being in the hyper-polypharmacy group. Several explanations are possible. Younger-old individuals may be in earlier, more intensive phases of chronic disease management, leading to more aggressive pharmacotherapy, while some older-old individuals might have undergone medication rationalization or deprescribing as part of geriatric care. Alternatively, survival bias may play a role: those who tolerate heavy medication regimens may be over-represented in the younger segment, whereas frailer older patients with high medication burden may be under-represented due to mortality.

An additional consideration is our operational definition of medication burden, which counted both prescription medicines and non-prescribed products (OTC medicines, vitamins/supplements, and herbal/traditional remedies). Differences in self-medication behavior by age group could therefore contribute to the observed association between younger age and hyper-polypharmacy. Future analyses that separate prescription-only medication counts from non-prescribed products-and that examine the composition of medication lists by age-may help clarify whether this pattern is driven primarily by prescribing intensity, self-medication, or both.

Regardless of the mechanism, our findings suggest that high medication burden is not simply a function of chronological age but is closely tied to functional status and multimorbidity. This supports individualized, rather than age-based, approaches to medication review and deprescribing.

### 4.5. Falls, Fear of Falling, and Polypharmacy

Falls and fear of falling were common in this cohort, with 87% reporting a history of falls and 41% having fallen in the past year. This aligns with literature linking polypharmacy to increased risk of falls and fractures [13,14,17]. In our multivariable analysis, however, history of falls did not emerge as an independent predictor of higher medication burden after adjusting for age, comorbidities, and function. This suggests that the relationship between falls and polypharmacy may be mediated by factors such as frailty and functional impairment rather than directly driven by the number of medications alone.

From a clinical perspective, this underscores the need to consider medication review as a standard component of multifactorial fall-prevention programs, especially for individuals with known falls and functional limitations.

## 5. Conclusions and Clinical Implications

In this cross-sectional study of Emirati community-dwelling older adults attending primary health centers in Ras Al Khaimah, we found a high prevalence of polypharmacy and hyper-polypharmacy, substantial multimorbidity, and extensive use of non-prescribed products. Higher medication burden was strongly associated with greater dependence in both basic and instrumental activities of daily living, and younger age and poorer functional status were independently associated with a greater likelihood of hyper-polypharmacy. This degree of medication burden is potentially harmful, given established links between polypharmacy and adverse drug reactions, drug–drug interactions, falls, hospitalizations, and mortality.

Although causality cannot be inferred from this design, our findings suggest that older adults with multimorbidity and functional limitations represent a particularly vulnerable group for high medication burden. These results support the routine integration of structured, patient-centered medication review and deprescribing initiatives into primary care and home-care services for older adults in the UAE.

Clinically, regular, comprehensive medication reviews ideally co-led by physicians and clinical pharmacists should be implemented to:

- Identify and discontinue non-essential or high-risk medications (including fall-risk-increasing drugs where possible).
- Reconcile prescription and non-prescribed products (OTC medicines, vitamins/supplements, and herbal/traditional remedies) to identify duplication and interactions.
- Consider non-pharmacological alternatives where appropriate.
- Prioritize medications that maintain function, mobility, and quality of life.

At the health-system level, incorporating deprescribing algorithms into national geriatric guidelines, embedding medication-review checkpoints into primary care workflows, and providing targeted training on geriatric pharmacotherapy and interprofessional collaboration will be essential. Public awareness campaigns could empower older adults and caregivers to engage in shared decision-making and to question unnecessary medications, especially non-prescribed ones.

## 6. Limitations and Recommendations

This study has several limitations. First, its cross-sectional design precludes causal inference; temporal relationships between medication burden, functional decline, and falls cannot be established. Second, the use of convenience sampling from two primary care centers limits generalizability to all older Emiratis in the UAE and may introduce selection bias. Third, we excluded older adults with moderate-to-severe cognitive impairment and those with severe physical dependence to ensure valid informed consent and reliable participation in the interview and medication verification process; this may have under-represented the most frail individuals, who may experience higher levels of polypharmacy and hyper-polypharmacy. Fourth, although we used a brown-bag review to verify medications, some OTC/traditional products may still have been underreported. Fifth, we did not formally assess prescribing appropriateness using tools such as the Beers Criteria or STOPP/START, and we did not evaluate off-label prescribing because medication indications were not systematically captured. Finally, we did not collect validated adverse drug reaction outcomes or link to pharmacovigilance/adverse-event registries; therefore, we could not directly quantify medication-related harm in this sample.

Future research should employ longitudinal designs to clarify the temporal relationship between medication burden and functional trajectories and to assess clinical outcomes such as falls, hospitalizations, and mortality. Studies should also separate prescription-only medication counts from non-prescribed products to quantify the contribution of self-medication and supplements, and to assess whether predictors differ by medication source. Incorporating explicit appropriateness criteria (e.g., Beers, STOPP/START) and documenting medication indications would enable evaluation of potentially inappropriate and off-label prescribing. Finally, linking primary-care medication data with national pharmacovigilance/adverse-event registries could help characterize adverse drug reactions and clinically important drug–drug interactions in older Emirati populations.

## Figures and Tables

**Table 1:** Demographic data of the study participants.

Parameter	Category	Study Participants (n = 200)
Gender	Male	84 (42.0%)
	Female	116 (58.0%)
Age (years)	Mean ± SD	74.3 ± 6.62
Age category	60 to 74	88 (44.0%)
	75 to 84	100 (50.0%)
	≥85	12 (6.0%)
Marital status	Single	10 (5.0%)
	Married	146 (73.0%)
	Widow	20 (10.0%)
	Divorced	24 (12.0%)
Educational level	Bachelor’s degree	46 (23.0%)
	Primary school	130 (65.0%)
	Read and write only	24 (12.0%)
Currently working	Yes	142 (71.0%)
Monthly income	Sufficient and save	40 (20.0%)
	Sufficient	20 (10.0%)
Living arrangement	Living with family	158 (79.0%)
	Alone	42 (21.0%)
Current smoker	Yes	56 (28.0%)
Life satisfaction (yes)	Yes	84 (42.0%)
Perceived health status	Poor	8 (4.0%)
	Regular	134 (67.0%)
	Good	34 (17.0%)
	Excellent	24 (12.0%)

Perceived health status is summarized in Table 1.

**Table 2:** Medical History in study participants.

Parameter	Study Participants (n = 200) n (%)
Suffering from chronic disease	180 (90.0%)
Diabetes Mellitus	170 (85.0%)
Orthopedic Diseases	166 (83.0%)
Kidney Diseases	154 (77.0%)
Cardiovascular Diseases	138 (69.0%)
Neoplasm	118 (59.0%)
Other	118 (59.0%)
Obesity	104 (52.0%)
Neuropsychiatric Problems	102 (51.0%)
Respiratory Diseases	72 (36.0%)

**Table 3:** Medication burden category and non-prescribed product use among study participants.

Parameter	Category	Study Participants (n = 200)
Medication burden category	Polypharmacy (5–9 medications)	120 (60.0%)
	Hyper-polypharmacy (≥10 medications)	80 (40.0%)
Non-prescribed products (OTC/supplements/herbal)	Any (≥1)	166 (83.0%)
	Analgesics	166 (83.0%)
	Vitamins/supplements	154 (77.0%)
	Laxatives	142 (71.0%)
	Other OTC/herbal/traditional	118 (59.0%)

Note: Non-prescribed product categories are not mutually exclusive; participants may report more than one type of non-prescribed product.

**Table 4:** Association between polypharmacy and Katz Index of Independence in study participants.

Parameter	Category	Polypharmacy (5–9 Medications) (n = 120)	Hyper-Polypharmacy (≥10 Medications) (n = 80)	*p*-Value
Bathing	Mean ± SD	1.00 ± 0.00	0.00 ± 0.00	<0.001
Dressing	Mean ± SD	0.92 ± 0.28	0.00 ± 0.00	<0.001
Toileting	Mean ± SD	1.00 ± 0.00	0.10 ± 0.30	<0.001
Transferring	Mean ± SD	1.00 ± 0.00	0.40 ± 0.49	<0.001
Continence	Mean ± SD	1.00 ± 0.00	0.40 ± 0.49	<0.001
Feeding	Mean ± SD	1.00 ± 0.00	0.45 ± 0.50	<0.001
Total Katz Index	Mean ± SD	5.92 ± 0.28	1.35 ± 1.60	<0.001
Total Katz Index grades	Independent	110 (91.7%)	0 (0.0%)	<0.001
Total Katz Index grades	Partially Dependent	10 (8.3%)	32 (40.0%)	<0.001
Total Katz Index grades	Totally Dependent	0 (0.0%)	48 (60.0%)	<0.001

**Table 5:** Association between polypharmacy and Lawton Scale for Instrumental Activities of Daily Living in study participants.

Parameter	Category	Polypharmacy (5–9 Medications) (n = 120)	Hyper-Polypharmacy (≥10 Medications) (n = 80)	*p*-Value
Ability to Use Telephone	Mean ± SD	0.80 ± 0.40	0.00 ± 0.00	<0.001
Shopping	Mean ± SD	0.98 ± 0.13	0.00 ± 0.00	<0.001
Food Preparation	Mean ± SD	1.00 ± 0.00	0.25 ± 0.44	<0.001
Housekeeping	Mean ± SD	1.00 ± 0.00	0.35 ± 0.48	<0.001
Laundry	Mean ± SD	1.00 ± 0.00	0.25 ± 0.44	<0.001
Mode of Transportation	Mean ± SD	1.00 ± 0.00	0.17 ± 0.38	<0.001
Responsibility for Medications	Mean ± SD	1.00 ± 0.00	0.30 ± 0.46	<0.001
Ability to Handle Finances	Mean ± SD	1.00 ± 0.00	0.45 ± 0.50	<0.001
Total Lawton Scale	Mean ± SD	7.78 ± 0.45	1.77 ± 2.40	<0.001
Lawton Scale total grades	Independent	120 (100.0%)	14 (17.5%)	<0.001
Lawton Scale total grades	Partially Dependent	0 (0.0%)	14 (17.5%)	<0.001
Lawton Scale total grades	Totally Dependent	0 (0.0%)	52 (65.0%)	<0.001

**Table 6:** Firth penalized logistic regression of predictors of hyper-polypharmacy (≥10 medications) versus polypharmacy (5–9 medications).

Predictor	Odds Ratio (OR)	95% Confidence Interval	*p*-Value
Age category (per category increase)	0.34	0.15–0.78	0.011 *
Katz ADL total score (0–6)	0.07	0.01–0.43	0.004 *
Gender (male vs. female)	1.11	<0.01–2.48	0.972
Marital status (not married vs. married)	0.78	0.01–1.81	0.911
Educational level (read/write only vs. primary/bachelor)	1.16	0.68–3.01	0.218
History of falls (yes vs. no)	1.04	0.01–1.97	0.987
Smoking status (current smoker vs. non-smoker)	1.05	0.89–2.98	0.289
Any chronic disease (yes vs. no)	0.45	<0.01–2.68	0.822
Respiratory disease (yes vs. no)	1.87	0.48–4.22	0.118
Cardiovascular disease (yes vs. no)	0.89	0.01–3.24	0.455
Kidney disease (yes vs. no)	0.03	<0.01–2.69	0.980
Neuropsychiatric problems (yes vs. no)	1.23	0.05–1.50	0.902
Any non-prescribed product use (yes vs. no)	0.003	<0.01–2.92	0.097
Vitamins/supplements use (yes vs. no)	0.18	<0.01–1.15	0.430
Laxatives use (yes vs. no)	1.65	0.02–2.03	0.816

OR = Odds Ratio; * = Statistically significant at *p* < 0.05.

## Data Availability

The data presented in this study are available on request from the corresponding author due to privacy and ethical restrictions.

## Supplementary Materials

The following supporting information can be downloaded at: https://www.mdpi.com/article/10.3390/healthcare14050648/s1, File S1: English version of the study questionnaire.
