# Supplementary material for: Prevalence and Associated Factors of Polypharmacy Among Emirati Community-Dwelling Older Adults: A Cross-Sectional Survey
Source: Healthcare (Basel). 2026 Mar 4;14(5):648. doi: 10.3390/healthcare14050648 (PMC12984632; doi:10.3390/healthcare14050648)
Supplement: Supplementary file 1 [file healthcare-14-00648-s001.zip › healthcare-4114953-supplementary.pdf]

Part I: Sociodemographic characteristic

Sociodemographic characteristic of elderly:

1- **Gender** 1- Male 2- Female
2- **Age............**
3- **Marital Status** 1- Single 2- Married 3- Widow 4- Divorced
4- **Educational level:**
 1- Read and write only 2- Primary school
 3-Bachelor degree 4- Post graduate

5- Are you working now 1- yes 2- No

6- Monthly income 1- Sufficient 2- Non-sufficient 3- Sufficient and save

7-With whom do you live?

1- Alone 2- Living with family

8-Do you Smoke? 1- yes 2- No

9- Are you satisfied with your life? 1- yes 2- No

10- What is your perception about your health? (poor, regular, good or excellent)

11- **Are you suffering from any of the following chronic diseases?**

1. Nil
2. Obesity ( )
3. Respiratory diseases ( )
4. Cardiovascular diseases ( )
5. Hypertension ( )

6.	Kidney diseases	( )
7.	Diabetes Mellitus	( )
8.	Orthopedic diseases	( )
9.	Neuropsychiatric problems	( )
10.	Liver disease	
11.	Neoplasm	( )
12.	Others…. Mention	( )

12- History of falls 1- yes 2- No

13- last fall < 12 months 1- yes 2- No

14- Fear of fall 1- Afraid 2- Not afraid.

Part II:

1. How many medications do you take per day?

A. Polypharmacy (≥5 medications) yes no;

B. Hyper polypharmacy (≥10 medications). yes no;

1. Do you use any non-prescribed drug? Yes No
2. If yes what is the type? Analgesic Vitamins Laxatives Others……….

Part III: Katz Index of Independence in Activities of Daily Living

**Activities**	**Independence**<br>(1 Point)<br>**NO** supervision, direction or	**Dependence**<br>(0 Points)<br>**WITH** supervision

	personal assistance.	,direction, personal assistance or total care.
**Bathing**	Bathes self completely or needs help in bathing only a single part of the body such as the back, genital area or disabled extremity.	Need help with bathing more than one part of the body, getting in or out of the tub or shower. Requires total bathing
**Dressing**	Get clothes from closets and drawers and puts on clothes and outer garments complete with fasteners. May have help tying shoes.	Needs help with dressing self or needs to be completely dressed.
**Toileting**	Goes to toilet, gets on and off, arranges clothes, cleans genital area without help.	Needs help transferring to the toilet, cleaning self or uses bedpan or commode.
**Transferring**	Moves in and out of bed or chair unassisted. Mechanical transfer aids are acceptable	Needs help in moving from bed to chair or requires a complete transfer.
**Continence**	Exercises complete self control over urination and defecation.	Is partially or totally incontinent of bowel or bladder
**Feeding**	Gets food from plate into mouth without help. Preparation of food may be done by another person.	Needs partial or total help with feeding or requires parenteral feeding.

Part IV: Lawton Scale

The Lawton Instrumental Activities of Daily Living Scale

A. Ability to Use Telephone

1. Operates telephone on own initiative; looks up and dials numbers 1
2. Dials a few well-known numbers 1
3. Answers telephone, but does not dial 1
4. Does not use telephone at all 0

B. Shopping

1. Takes care of all shopping needs independently 1
2. Shops independently for small purchases 0
3. Needs to be accompanied on any shopping trip 0
4. Completely unable to shop 0

C. Food Preparation

1. Plans, prepares, and serves adequate meals independently 1
2. Prepares adequate meals if supplied with ingredients 0
3. Heats and serves prepared meals or prepares meals but does not maintain adequate diet 0
4. Needs to have meals prepared and served 0

D. Housekeeping

1. Maintains house alone with occasion assistance (heavy work) 1
2. Performs light daily tasks such as dishwashing, bed making 1
3. Performs light daily tasks, but cannot maintain acceptable level of cleanliness 1
4. Needs help with all home maintenance tasks 1
5. Does not participate in any housekeeping tasks 0

E. Laundry

1. Does personal laundry completely 1
2. Launders small items, rinses socks, stockings, etc 1
3. All laundry must be done by others 0

F. Mode of Transportation

1. Travels independently on public transportation or drives own car 1
2. Arranges own travel via taxi, but does not otherwise use public transportation 1
3. Travels on public transportation when assisted or accompanied by another 1
4. Travel limited to taxi or automobile with assistance of another 0
5. Does not travel at all 0

G. Responsibility for Own Medications

1. Is responsible for taking medication in correct dosages at correct time 1
2. Takes responsibility if medication is prepared in advance in separate dosages 0
3. Is not capable of dispensing own medication 0

H. Ability to Handle Finances

1. Manages financial matters independently (budgets, writes checks, pays rent and bills, goes to bank); collects and keeps track of income 1
2. Manages day-to-day purchases, but needs help with banking, major purchases, etc 1
3. Incapable of handling money 0

Scoring: For each category, circle the item description that most closely resembles the client's highest functional level (either 0 or 1).

الجزء الأول :الخصائص الاجتماعية والديموغرافية

الخصائص الاجتماعية والديموغرافية لكبار السن:

1. الجنس: ذكر أنثي
2. العمر.........
3. الحالة الاجتماعية - أعزب - 2 متزوج - 3 أرمل - 4 مطلق
4. المستوى التعليمي

1- القراءة والكتابة فقط - 2 المدرسة الابتدائية

- 3بكالوريوس - 4 دراسات عليا

- 5هل تعمل الآن - 1 نعم - 2 لا

- 6الدخل الشهري 1 - كافي - 2 غير كافي 3 - كافي وآمن

- 7مع من تعيش ؟

1- وحدك - 2 العيش مع العائلة

هل تُدَخِن؟ - 1نعم 2- لا

- 9هل أنت راضٍ عن حياتك ؟ - 1 نعم 2- لا

- 10ما هو تصورك عن صحتك ؟) ضعيف، منتظم، جيد أو ممتاز(

- 11هل تعاني من أي من الأمراض المزمنة التالية ؟

1. لايوجد
2. السمنة ()
3. أمراض الجهاز التنفسي
4. أمراض القلب والأوعية الدموية
5. ارتفاع ضغط الدم ()

6. أمراض الكلى ()
7. داء السكري ()
8. أمراض العظام
9. مشاكل نفسية وعصبية
10. Liver disease
11. أورام ()
12. أخرى اذكر ()

- **12**تاريخ السقوط - 1 نعم - 2 لا

- **13**الخريف الماضي **12** > شهرًا - 1 نعم - 2 لا

- **14**الخوف من السقوط - **1** الخوف - 2 عدم الخوف.

الجزء**:II**

1.كم عدد الأدوية التي تتناولها يوميًا ؟

أ. تعدد الأدوية (أكبر من أو يساوي5 أدوية) نعم لا ؛

ب .فرط تعدد الأدوية (أكبر من أو يساوي10 أدوية). نعم لا ؛

1. هل تستخدم أي دواء غير موصوف ؟ نعم لا
2. إذا كانت الإجابة بنعم، فما هو النوع ؟ فيتامينات مسكنة ملينات أخرى..........

الجزء الثالث استخدام مقياس لوتن لتقييم أنشطة الحياة اليومية الأدائية

أنشطة الحياة اليومية	قادر (2)	غير قادر (1)
القدره علي استخدام التليفون		

الذهاب للتسوق		
اعداد الطعام		
تنظيف المنزل		
القيام بالغسيل		
استخدام المواصلات والتنقل من مكان لاخر		
تناول الادوية		
القدره علي التعاملات الماليه		
**المجموع**		

المجموع (16) للسيدات و (10) للرجال وتقسم علي 100%

- 25% يعتمد كليا علي الأخرين
- 25-75% يحتاج مساعدة

أكثر من 75% لايعتمد علي أحد

مقياس كاتز الخاص بمدى الاعتماد على النفس فى أنشطة الحياة اليومية

**أ- الحمام- سواء كان الحمام فى البانيو- الدوش- حمام ناشف**		
(1)	(2)	(3)
لا يساعدة أحد (يدخل ويخرج من البانيو بنفسة اذا كان البانيو هو المعتاد للحمام)	يحتاج مساعدة فى الاستحمام لجزء واحد من أجزاء جسمة مثل الظهر أو الرجل	يعاونة أحد فى عملية الاستحمام لأكثر من جزء فى الجسم
**ب-الملبس-يحضر الملابس من الدولاب أو من الدرج بما فى ذلك الملابس الداخلية- الثياب الخارجية ويستخدم أشياء للتثبيت مثل:**		
(1)	(2)	(3)
يلبس ملابس كاملة أو الفستان بدون مساعدة أى شخص	يلبس ملابسة أو الفستان بدون مساعدة أحد ما عدا إحتياجة لأى شخص فى ربط الحذاء	يساعدة أحد فى لبس ملابس كاملة لبس الفستان أو يظل بدون لبس كامل أو نصف لبسة
**ج-الذهاب للتواليت-يذهب للحمام –حجرة التواليت لعمل البول والبراز-ينظف نفسة بعد عملية الاخراج ويعيد انزال ملابسة أو جعلها مرتبة بعد عملية الاخراج**		
(1)	(2)	(3)
يذهب للحمام(حجرة التواليت) ينظف نفسة ويعيد ارجاع ملابسة بدون مساعدة أى شخص (مستعينا باستخدام أشياء مثل: المشاية- الكرسى المتحرك) ويمكن تنظيف وتفريغ ما أستخدم مساء فى عملية الاخراج :مثل تنظيف القعادة بجانب السرير	يحتاج لمساعدة فى الذهاب للتواليت أو فى تنظيف نفسة أو اعادة ترتيب ملابسة بعد عملية الاخراج أو فى استخدام القعادة مساء	لا يذهب للتواليت لعملية الاخراج
**د- التحرك أو التنقل**		
(1)	(2)	(3)

يتحرك داخل السرير وخارجة بدون مساعدة (يمكنة إستخدام أشياء كالعصا والمشاية لتحفظ توازنة)	يتحرك خارج وداخل السرير والكرسى بمساعدة شخص ما	لا يقوم من السرير مطلقا
**هـ-التحكم فى عملية الاخراج:**		
(1)	(2)	(2)
يتحكم فى عملية البول والبراز كاملة بارادتة	فى بعض الأحيان لا يتحكم فى البول والبراز	الاشراف أو المراقبة يساعدة على التحكم فى البول أو البراز هل سيستخدم قسطرة بول أو أنة لا يتحكم فى البول أو البراز
**و- الطعام**		
(1)	(2)	(3)
يطعم نفسة بدون مساعدة	يطعم نفسة ولكنة يحتاج مساعدة فى تقطيع اللحم أو دهن الخبز بالزبدة	يساعدة أحد فى عملية الطعام كاملة أو نصف كاملة أما باستخدام أنابيب كأنبوبة المعدة أو عن طريق المحاليل

مجموع النقاط (18):

- 13الي 18 يعتمد كليا علي الاخرين
- 7 الي 12 يحتاج مساعدة للقيام
- 6 يعتمد علي نفسه
